# Design of Ni(OH)_2_ Nanosheets@NiMoO_4_ Nanofibers’ Hierarchical Structure for Asymmetric Supercapacitors

**DOI:** 10.3390/nano12224079

**Published:** 2022-11-19

**Authors:** Junzhu Li, Xin Chang, Xuejiao Zhou, Mingyi Zhang

**Affiliations:** Key Laboratory for Photonic and Electronic Bandgap Materials, Ministry of Education, School of Physics and Electronic Engineering, Harbin Normal University, Harbin 150025, China

**Keywords:** Ni(OH)_2_, NiMoO_4_, nanofibers, electrospinning, supercapacitor

## Abstract

Transition−metal−based materials show great promise for energy conversion and storage due to their excellent chemical properties, low cost, and excellent natural properties. In this paper, through simple strategies such as classical electrospinning, air calcination, and the one−step hydrothermal method, a large area of Ni(OH)_2_ nanosheets were grown on NiMoO_4_ nanofibers, forming NiMoO_4_@Ni(OH)_2_ nanofibers. The one−dimensional nanostructure was distributed with loose nanosheets, and this beneficial morphology made charge−transfer and diffusion more rapid, so the newly developed material showed good capacitance and conductivity. Under the most suitable experimental conditions, the optimal electrode exhibited the highest specific capacitance (1293 F/g at 1 A/g) and considerable rate capability (56.8% at 10 A/g) under typical test conditions. Most interestingly, the corresponding asymmetrical capacitors exhibited excellent electrochemical cycle stability, maintaining 77% of the original capacitance. NiMoO_4_@Ni(OH)_2_ nanofibers were verified to be simple to prepare and to have good performances as energy−storage devices within this experiment.

## 1. Introduction

As a new kind of high−power−density device, supercapacitors can achieve a fast charge and discharge within a few seconds, which show their excellent electrochemical abilities, excellent cycle performances, and high ratio discharge properties. This has far−reaching implications for the progress of electrochemical energy storage. Supercapacitors that combine the advantages of chemical batteries and electrostatic capacitors are more utilized in the market. As we all know, with the development of technology, the variety of electrode materials is constantly being enriched, so the mechanisms of their electrical storage are also being studied more and more. Different electrode materials are classified as different reaction mechanisms, some of which reflect the superior performance of multiple energy−storage mechanisms under the combined action. From the perspective of material classification, it can be divided into three types: (1) carbon−based materials with large specific surface areas [1], (2) conductive polymer materials with good pseudo−capacitances, and (3) metal composites with high theoretical capacitances. According to the above information, a carbon material is used as electrostatic energy−storage material, and its charge adsorption and desorption occur at the interface of the carbon nanomaterial electrode and electrolyte. Conductive polymers and metallic compounds are pseudo−capacitive materials in which material energy can be stored through Faraday reactions.

Over the years, the main areas of research, such as transition−metal compounds as electrodes for supercapacitors, have been applied on a large scale due to the excellent chemical properties, electronic properties, and diverse chemical states. Transition metal oxides (TMOs) have the characteristics of simple preparation methods and easy control of nanostructures. However, there are still many problems with TMOs, such as their magnification performances, poor cycle stabilities, high preparation costs, and high toxicities, which lead to their limitation in practical applications. The researchers found that spinel binary transition metal oxides have better conductivity than single−transition metal oxides, theoretically greater than larger capacitives and richer centers of electrochemical activity. Tarasankar and the group successfully prepared NiCo_2_O_4_ nanosheets with a thinner thickness (less than 20 nm) [2], which was conducive to the rapid occurrence of surface redox reactions and could provide more active sites. Measured as 1540 F/g at a current density of 1 A/g, the specific capacitance could still reach 89% of the initial capacitance after a continuous 10,000 cycles at a current density of 30 A/g [3]. Wang et al. synthesized FeCo_2_O_4_−based composites with core/shell nanowire structures on carbon cloth by hydrothermal synthesis. The asymmetric capacitors consisted of samples as positive electrodes and activated carbon as negative electrodes; they had excellent flexibility and output a maximum energy density of up to 94.9 Wh·kg^−^^1^ [4]. As the active material of the supercapacitor, pure−phase monoclinic NiMoO_4_ had a discharge capacitance of 815 F/g **[5]**. However, as molybdenum has recently faced a global supply shortage and its price has increased compared with the past, electrostatic spinning has been used to construct one−dimensional NiMoO_4_ nanofibers in order to reduce costs. Electrostatic spinning is a convenient and versatile emerging technology that allows for the construction of a relatively large number of fabric nanostructures with various morphologies from a small amount of raw material. One−dimensional nanofibers constructed by electrostatic spinning have been shown by many researchers to have excellent electrocatalytic properties, such as hydrogen (HER) or oxygen (OER) production from water−cracking under acidic or alkaline conditions [6,7,8,9,10]. At the same time, the large specific surface area of 1D nanofibers allows them to bind more easily to other compounds. Layered compounds such as Ni(OH)_2_ nanosheets can be grown on the surface of a one−dimensional (1D) material [11], making this a method of changing the properties of a material by regulating the shape of a structure. Covering Ni(OH)_2_ nanosheets on a 1D material has following advantages; (i) The size of the nanosheets may affect the capacitance properties of multi−recombinant materials. Therefore, by changing the ratio of the reactants to control the nanosheets’ growth allows for the formulation of the most suitable size. (ii) Large swathes of nanomaterials grow directly into the crosslinked fiber mesh, which enhances conductivity and also helps to reduce the length of the ion diffusion path at the electrode/electrolyte interface. The combination of the two nanostructures expands the reaction area, provides a fast channel for fast electron transport, and avoids the aggregation of multilayer structures, ultimately enhancing the electrochemically active site of the entire electrode material. The feasibility of growing hydroxide nanosheets on nickel foam to prepare supercapacitors **[12]** and of wrapping nickel hydroxide around nickel molybdate nanorods has been reported by researchers **[13]**.

Based on these considerations, this study reports a simple, straightforward, and efficient method for the synthesis of one−dimensional linear nickel molybdate nanofibers encapsulated in nickel molybdate nanosheets which requires only trace amounts of raw materials in order to reduce costs while achieving a balance between reducing materials and maintaining excellent properties. One−dimensional Ni(OH)_2_ nanosheet composite NiMoO_4_ nanofibers for supercapacitor electrodes are synthesized by classical electrostatic spinning, air calcination, and the one−step hydrothermal method. In this study, the electrochemical performances of the prepared NiMoO_4_@Ni(OH)_2_ nanofibers were systematically studied [14], and asymmetric supercapacitors were assembled to explore their capacitive properties as practical applications. Indeed, the NiMoO_4_@Ni(OH)_2_ nanofibers electrode material exhibited an excellent specific capacitance of 1293 F/g at 1A/g, while saving 60.8% of the virginal capacitance after 5000 cycles. Furthermore, the rate capability obtained was almost 56.8% of that at the beginning. The two−electrode asymmetric supercapacitor (ASC) assembled by NiMoO_4_@Ni(OH)_2_ nanofibers and an activated carbon (AC) device exhibited the energy density of 46.8 Wh/kg at 700 W/kg and an excellent capacitance ratio (77% after 5000 cycles). It is worth noting that in practical applications, the ASC device successfully illuminated the light plate with 23 red−light−emitting diodes, indicating that the NiMoO_4_@Ni(OH)_2_ nanofibers have great potential for practical applications.

## 2. Experimental Section

### 2.1. Materials

Polyacrylonitrile (PAN, Mw ~150,000) was purchased from Sigma−Aldrich Corporation. *N*,*N*−dimethylformamide (DMF), Ni(CH_3_COO)_2_·4H_2_O, hexamethylenetetramine (HMTA), and (NH_4_)_6_Mo_7_O_24_·4H_2_O were prepared from Zhiyuan Reagent (Tianjin Kaida Chemical Plant, Tianjin, China).

### 2.2. Synthesis of NiMoO_4_ Nanofibers

The NiMoO_4_ nanofibers were synthesized according the previously described method [15]. To prepare the NiMoO_4_ nanofibers, 500 mg PAN powder was dissolved in 5 mL DMF and stirred for 1 h at room temperature to form a transparent solution. A total of 0.7 mmol Ni(CH_3_COO)_2_·4H_2_O and 0.1 mmol (NH_4_)_6_Mo_7_O_24_·4H_2_O were orderly added to the above mixture and stirred for 12 h to obtain a clear solution. The solution was then poured into a 20 mL adapter for the next step. In order to stabilize the electrostatic spinning process, the operating voltage was set to 6 kV, and the distance between the adjustment needle and the receiver plate was 13 cm. With a certain downward slope, the solution could only flow out after breaking through the surface tension of the liquid, forming a solidified fiber mat on the tin foil after several hours of continuous operation. Afterwards, the resulting PAN/salt matrix was heated to 500 °C at a rate of 2 °C/min for 2 h under an air atmosphere to sustain the stable nanostructure. The sample was named NMO.

### 2.3. Synthesis of NiMoO_4_@Ni(OH)_2_ Nanofibers

For the preparation of the NiMoO_4_@Ni(OH)_2_ nanofibers, 0.06 mmol Ni(NO_3_)_2_•6H_2_O and 0.12 mmol HMTA were thoroughly mixed in 30 mL deionized water, then the mixed solution and 40 mg NMO sample were transferred to a Teflon−lined autoclave and heated at 90 °C for 8 h. The sample was washed with DI and ethanol to obtain the NiMoO_4_@Ni(OH)_2_ nanofibers, which were named NMH−1. Control samples were prepared by the above solvothermal method with different solution concentrations and a constant NMO sample. A total of 0.08 mmol and 0.16 mmol of Ni(NO_3_)_2_•6H_2_O and HMTA, respectively, formed a sample that was named NMH−2. The solution containing 0.10 mmol of Ni(NO_3_)_2_•6H_2_O and 0.20 mmol of hexamethylenetetramine was called NMH−3.

### 2.4. Characterizations

The morphologies and structures of the samples were characterized by scanning electron microscopy (SEM, Tokyo, Japan, Hitachi, SU70), transmission electron microscopy (TEM, FEI, Hillsboro, OR, USA, Tecnai TF20), and X-ray diffraction (XRD, Tokyo, Japan, Japan Institute of Technology, Rigaku D/max2600) analyses with a Cu Kα radiation source (λ = 0.154178 nm). In addition, the BET analysis was used to measure the specific surface areas of the samples.

### 2.5. Electrochemical Measurements

All of the OER and HER electrochemical performances were performed on a VMP3 electrochemical workstation with a three−electrode system in 1 M KOH electrolyte. A standard calomel electrode (SCE) and a graphite rod were used as the reference and counter electrodes, respectively. To prepare the working electrode, we mingled the nanofibers, the acetylene black, and polytetrafluoroethylene (PTFE) binder based on 8:1:1 weight proportion. Then, the mixture was printed on the carbon paper and dried for 24 h. The linear sweep voltammetry (LSV) curves were recorded with a scan rate of 5 mV s^−1^ with iR−compensation. The calculated potentials were all referred to as the reversible hydrogen electrode (RHE) obeying the relationship of E*_RHE_* = E*_SCE_* + 0.2415 V + 0.059*pH. Electrochemical impedance spectroscopy (EIS) tests were recorded in the frequency range from 0.01 Hz to 10^4^ Hz. The stability of electrocatalyst was evaluated by successive cyclic voltammetry tests. Double−layer capacitance (C*_dl_*) measurement roughly measured the electrochemical surface area (ESCA) through the linear relationships between the different scan rates and the current density. The turnover frequency (TOF) was calculated by scanning the surrounding areas of the cyclic voltammetry tests in a 1 M PBS solution.

The VMP3 (France) electrochemical test equipment was used to evaluate the properties of the as−prepared samples. The platinum sheet and the Ag/AgCl electrode set were used as the counter and reference electrodes, respectively [16]. The working electrode preparation process was to ground the required materials, conductive agents, and adhesives, coating on conductive substrate nickel foam. Then, the material was dried at 60 °C for 12 h in vacuum. Through the same process, the active carbon (AC) was smeared onto cut NF (0.5’0.5 CM^2^) for the preparation of another electrode. Cyclic voltammetry (CV), galvanostatic charge/discharge (GCD), and electrochemical impedance spectroscopy (EIS) tests were carried out in 2 M KOH [17].

The two−electrode measurement was also performed in 2 M KOH. The asymmetric capacitors (ASC) equipment was assembled with the NiMoO_4_@Ni(OH)_2_ nanofibers and active carbon as the positive and negative electrodes, respectively [18]. The load of the activated carbon electrode could be set by the charge balance theory, and the charge storage amount could be obtained according to the calculation formula Equations (1) and (2). *C*, Δ*V*, *I*, and m in the equations represent the specific capacitance, voltage range, current density, and loading mass, respectively [19,20,21,22,23].

(1) $$C_{s}=\frac{I\times t}{\Delta V\times m}$$

(2) q=C×ΔV×m

Δ*t* and *C_s_* correspond to the discharge time and the specific capacitance that exist in the above equation, respectively. To ensure the q+ = q−, we used Equation (3) [24].

(3) $$m_{+}C_{+}V_{+}=m_{-}C_{-}V_{-}$$

As mentioned in the formula, +/− represent the electrodes on both sides of ASC device, respectively. The optimum ratio of $m_{+}$/$m_{-}$ for the device was 0.2. The energy and power density could be obtained by the following calculation Equations (4) and (5) [25,26,27,28].

(4) $$E=\frac{{\mathrm{CV}}^{2}}{2}$$

(5) $$P=\frac{3600E}{\Delta t}$$

## 3. Results and Discussion

**Scheme 1:**
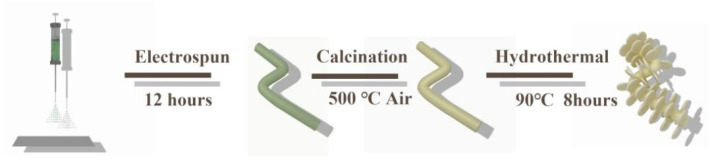
Schematic diagram showing the construction of the NiMoO_4_@Ni(OH)_2_ nanofibers.

**Figure 1:**
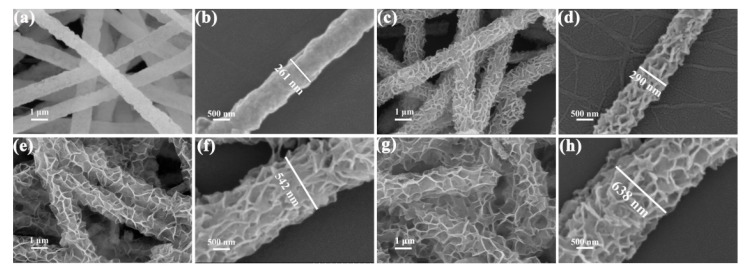
SEM images of (**a**,**b**) NMO, (**c**,**d**) NMH−1, (**e**,**f**) NMH−2, and (**g**,**h**) NMH−3.

Scheme 1 shows the synthesis process of NMH. Firstly, the NiMo/PAN nanofibers were prepared through the classical electrospun method. Afterwards, the NiMoO_4_ nanofibers (NMO) were prepared by calcining above composite nanofibers in air [29]. Lastly, the NMH−X nanofibers were prepared by a one−step hydrothermal process. The microstructures and morphologies of the NiMoO_4_ nanofibers (NMO) and NiMoO_4_@Ni(OH)_2_ nanofibers−1,2,3 (NMH−1.2.3) were characterized by scanning electron microscopy (SEM). As shown in Figure 1a, the NMO presented well−organized structures with more spaces between each fiber. These NMO showed a neat and clear surface. As shown on a local SEM image of the NMO, the nanostructure size of the NMO was about 261 nm on average (Figure 1b). This ideal morphology may have been caused by the continuous and stable filament output during electrospinning. The template PAN was removed by the air during the calcination of the NiMo/PAN nanofibers, and then the metal oxide grains gradually coalesced inwards to form a solid one−dimensional structure [10]. After a typical hydrothermal process, the nanosheets of Ni(OH)_2_ were evenly distributed on the surface of the NMO and densely arranged in the network of fiber cross−linking, showing an obvious core/shell structure. In the low−magnification SEM image of NMH−1 (Figure 1d), the nanofibers covered by the nanosheets structure became larger that NMH−1 at about 290 nm on average. With the solution concentration adjustment during the hydrothermal process, the average diameter of NMH−2 was increased to almost 542 nm, and the nanosheets morphology of NMH−2 was relatively shown a small aggregation degree (Figure 1e,f). Due to the overgrowth of the Ni(OH)_2_ nanosheets, the surfaces of NMH−3 were denser than NMH−2, and the nanosheets were filled with spaces in the nanofibers network. Considering that the electrochemical reaction needed to be stretched and the reaction area required was wide, the morphology of MNH−2 was more suitable, so NMH−2 was suitably studied in relation to the electrochemical performance.

**Figure 2:**
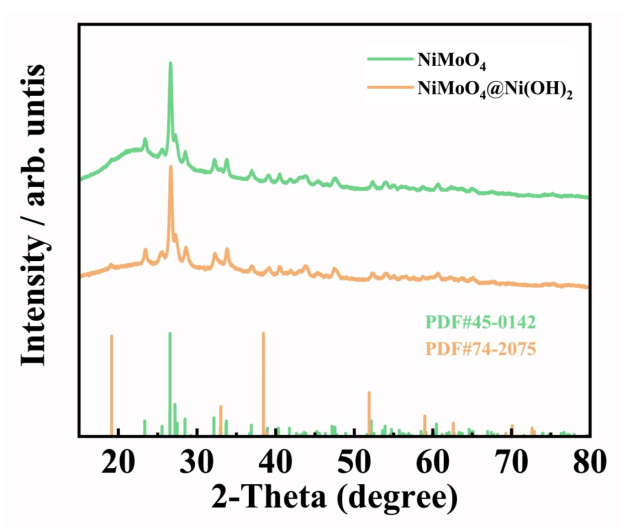
XRD patterns of the NiMoO_4_ and NiMoO_4_@ Ni(OH)_2_ nanofibers.

**Figure 3:**
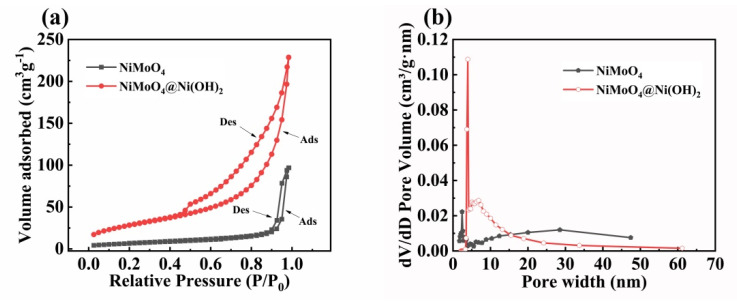
(**a**) N_2_ adsorption–desorption isotherms and corresponding (**b**) pore size distribution curves of the NMO and NMH−2.

The phase identification of the NiMoO_4_ and NiMoO_4_@ Ni(OH)_2_ nanofibers was formed by the XRD patterns in Figure 2. It can be seen from the NiMoO_4_ nanofibers that the characteristic diffraction peaks at 23.4°, 26.5°, 27.2°, 28.5°, and 32.1° perfectly assigned to the (0 2 −1), (002), (1 1 −2), (3 1 −1), and (112) crystallographic planes of the monoclinic NiMoO_4_ (PDF#45−0142), respectively, proving the success of the synthesis of NMO. It is obvious that the XRD result of the NiMoO_4_@ Ni(OH)_2_ nanofibers not only shows that the above peaks belong to monoclinic NiMoO_4_, but that in addition the characteristic diffraction peaks of Ni(OH)_2_ in the composite samples are not obvious. The main reasons may be as follows: first, the content of Ni(OH)_2_ was small, and the crystallization was poor. Secondly, the crystallization of the NiMoO_4_ prepared by calcination was better and the diffraction peak was stronger, which covered the diffraction peak of Ni(OH)_2_. We will conduct further analysis in the TEM test later. Combining the above analyses of SEM and XRD allowed for the further detection of the porosity and BET data of the samples via N_2_ physisorption. The sample exhibited typical mesoporous material characteristics, as shown in the Figure 3a; the isothermological linear type belongs to type IV, and it can be observed that NMH−2 has a larger specific surface area of about 179.2 m^2^/g compared with the precursor, which is consistent with the SEM characterization. The experimental isotherm in Figure 3a was accompanied by hysteresis, which may be due to the effect of capillary condensation on the isotherm [30]. In addition, the pore diameter center was about 3.9 nm, demonstrating that such a special material can provide more reaction areas for electrochemical reactions, therefore making them more suitable for charge transfer and diffusion.

**Figure 4:**
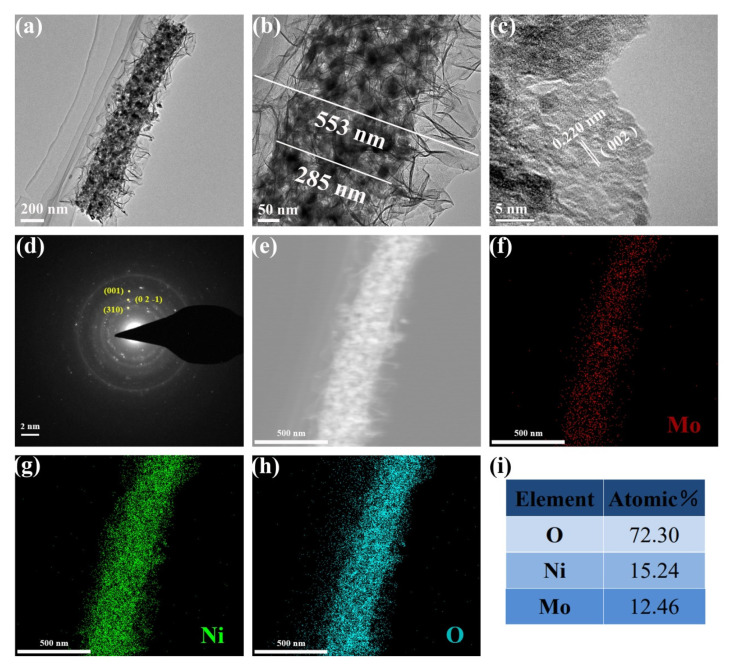
(**a**,**b**) TEM images, (**c**) HTEM image, (**d**) SAED pattern, (**e**) STEM, and (**f**–**h**) element mappings for Mo, Ni, and O of the NMH−2 and the (**i**) elemental contents of Mo, Ni, and O of NMH−2.

As shown in Figure 4, TEM was carried out to further reveal the finer nanostructures of the NMH−2 samples. The Ni(OH)_2_ nanosheets were wound around the surface of the NMO with a width of about 285 nm, so that the NMH−2 as a whole become coarser and larger in size. This morphology could offer a channel for electrons and ions (Figure 4a,b).The partial TEM image (Figure 4c) also depicts that the NMH−2 presented obvious lattice fringes with interplanar distances of 0.22 nm, which were ascribed to the (002) planes of Ni(OH)_2_. The selected area electron diffraction (SAED) pattern (Figure 4d) presented concentric rings, corresponding to the (310) and (0 −2 0) planes of NiMoO_4_ and the (001) planes of Ni(OH)_2_ from nanofibers to nanosheets. It clearly shows that the Ni, Mo, and O elements were uniform decorative in the sample, while the Mo element was located in the middle of sample, possessing nanosheets wholely covered on the surface of NMH−2 (Figure 4e–h). The EDS pattern (Figure 4i) of NMH−2 confirms the atomic percentages of Mo, Ni, and O are 12.46, 15.24, and 72.30%, respectively, which corresponds to the above data. The EDS pattern of all of the samples are shown in Table 1.

**Figure 5:**
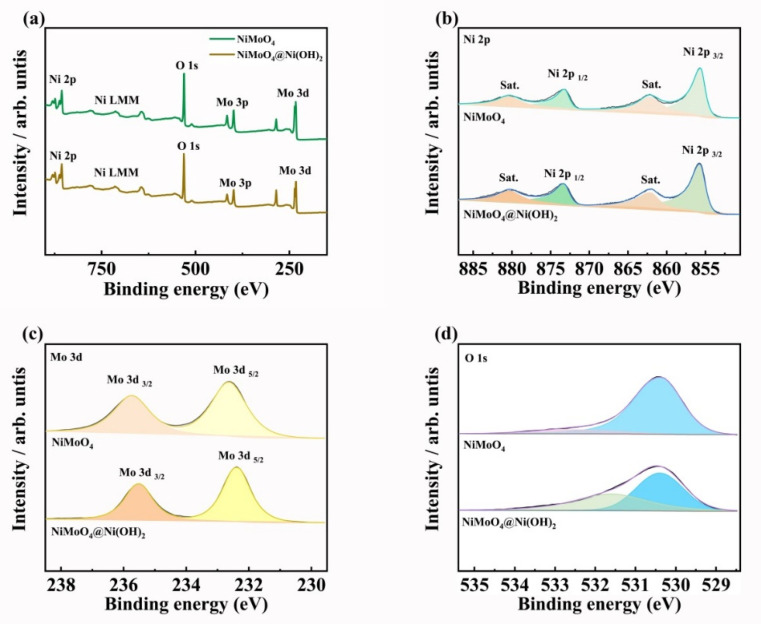
(**a**) XPS survey spectrum of NMO and NMH−2. (**b**) Ni 2p, (**c**) Mo 2p and (**d**) O 1s.

The chemical compositions and bonds were studied by XPS analysis. The XPS spectrums of NMO and NMH−2 are showed in Figure 5. As shown in Figure 5a, the XPS spectra of NMO and NMH−2 confirmed the relevant elements present in the sample, matching the result of above analysis. The Ni peaks of NMO were located at 855.6 and 873.1 eV, which match the Ni 2p_3/2_ and Ni 2p_1/2_. The binding energies of the Ni species satellite peaks at 862.0 and 880.3 eV, respectively (Figure 5b) [31,32,33], are also shown. In addition, the difference between the Ni 2p_3/2_ and Ni 2p_1/2_ peaks was measured at about 17.5 eV, indicating that divalent nickel species were present in the sample [34]. Compared with NMO, the peaks of Ni 2p_3/2_ and Ni 2p_1/2_ for NMH−2 shifted by ~0.1 eV towards higher binding energy. As shown in Figure 5c of Mo 3d in two samples, the peaks located at 232.3 and 235.5 eV can be assigned to Mo 3d_5/2_ and Mo 3d_3/2_, respectively, which correspond to Mo^6+^ [35]. By further analyzing the peaks of NMO and NMH−2, it was found that the hydrothermal process did not affect the valence state of Mo [36]. However, with the decrease of the Mo proportion, the Mo 3d_5/2_ peak of the final product gradually shifted to a lower binding energy. From Figure 5d, two peaks in the NMO can be seen. The peaks at 530.2 and 532.5 eV correspond to lattice oxygen−metal (M−O) on the material surface and hydroxyl groups or surface−adsorbed oxygen, respectively [35,37]. Furthermore, two peaks were present in the NMH−2. Specifically, the two peaks located at 530.3 eV and 531.5 eV in O 1’s core level spectrum indicate the existence of metal−oxygen bonds and OH groups, respectively [38,39]. In addition, The XPS test of NMH−2 confirms that the atomic percentages of Mo, Ni, and O are 12.70, 16.17, and 71.13%, respectively, which correspond to the above EDX data. The above analysis shows that NMO and NMH−2 were successfully synthesized.

**Figure 6:**
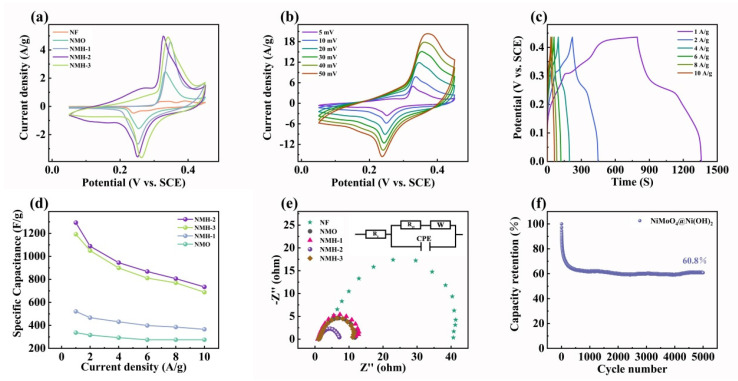
(**a**) CV curves of the NF, NMO, NMH−1, NMH−2, and NMH−3 at 5 mV/s. (**b**) CV curves of the NMH−2 at 5–50 mV/s. (**c**) GCD curves of the NMH−2 at 1–10 A/g. (**d**) Specific capacitances of the NMO, NMH−1, NMH−2, and NMH−3 at 1–10 A/g. (**e**) Nyquist plots of the NF, NMO, NMH−1, NMH−2, and NMH−3. (**f**) Cyclic performance of the NMH−2 at 10 A/g.

The electrochemical behaviors of NF, NMO, and NMH−1,2,3 were determined in the three−electrode system in 2 M KOH. These electrodes were measured via CV, GCD, and EIS. As shown in Figure 6a, the potential window set from 0.05 to 0.45 V with the scan rate of 5 mV s^−1^ of all the above materials, and the NMH−2 displays the largest integrated area of the CV curve, which might be superior due to the electrode reaction on the surface acting fast and continuing, resulting the highest capacitance. Specifically, a distinct pair of redox peaks were observed, mainly governed by the Faradaic redox reactions of Ni (II) ↔ Ni (III) + e^−^. Faradic redox reactions are as follows [40,41,42,43,44]:

(6) $${\left[\mathrm{Ni}{\left(\mathrm{OH}\right)}_{3}\right]}^{-}↔\mathrm{NiO}+H_{2}O+{\mathrm{OH}}^{-}$$

(7) $$\mathrm{NiO}+{\mathrm{OH}}^{-}↔\mathrm{NiOOH}+e^{-}$$

The NMH−2 electrodes exhibited larger redox peaks than the other samples, which implies a superior pseudocapacitive behavior of NMO. The Ni(OH)_2_ nanosheets grown on the surface of NMO considerably increased the carrier density and enhanced the surface redox reaction reactivity of NMO. Furthermore, as Figure 6b shows, the CV curves of NMH−2 can remain undistorted within the scan rates as they increase, implying that the electrochemical process is highly reversible and demonstrating the excellent capacitance exhibited in each curve. In Figure 6c, the galvanostatic charge/discharge voltage profiles of NMH−2 at 1 mA cm^−2^ can be seen, along with the great discharge time of NMH−2 was 564 s and the wonderful performance of its energy storage. As presented in Figure 6d, the specific capacitances of the electrode materials were estimated. At the current density of 1 A/g, the specific capacitance of NMH−2 was 1293 F/g, which exceeds that of other samples such as NMO (337 F/g), NMH−1 (521 F/g), and NMH−3 (1192 F/g). At a high current density of 10 A/g, the NMH−2 still maintained 734 F/g, demonstrating a superior rate capability (56.8%). The higher the current density, the shorter the electrochemical reaction time, and the lower the capacitance performance exhibited. The superior behaviors of NMH−2 in the electrochemical test might be related to the synergy reaction of the electrode. Furthermore, the Faraday reaction on the electrode surface was evaluated by by EIS. The Nyquist plots of nickel foam, NMO, NMH−1, NMH−2, and NMH−3 show the standard shape in the high−frequency zone (Figure 6e). In addition, the equivalent series resistance (Rs) can be calculated by interception of the Z′−axis, and the diameter distance in the impedance spectrum matches the transfer charge resistance (Rct). It is clearly shown that the electrochemical impedance spectroscopy of the NMH−2 showed the lowest values of Rs and Rct (0.63 and 0.1 Ω, respectively) among these catalysts. More practically, cycling durability is the most crucial factor for the comment of supercapacitors. As can be seen from Figure 6f, NMH−2 have shown an excellent cycling ability after long−term testing (5000 cycles) at a high current density of 10 A/g, and could still maintain 60.8% of their original performance compared with the initial. The improved electrochemical property of the NMH−2 is ascribed to the following factors. First, the Ni(OH)_2_ nanosheets provided the bigger active surface area compared with pure NMO for testing capacitance, and the crosslinked nanofibers captured OH^−^ in the electrolyte. Second, the 1D NMO precursor could serve as a template to link the nanosheets for accelerating the transport of the two components. Finally, the synergistic effect between the NMO and Ni(OH)_2_ nanosheets promoted an improved and stable capacitance performance. At the same time, we evaluated some recent research results and compared the charge storage performance of various related supercapacitors with that of the prepared NMH−2. The results show that NMH−2 had relatively good properties under alkaline conditions (Table 2).

**Figure 7:**
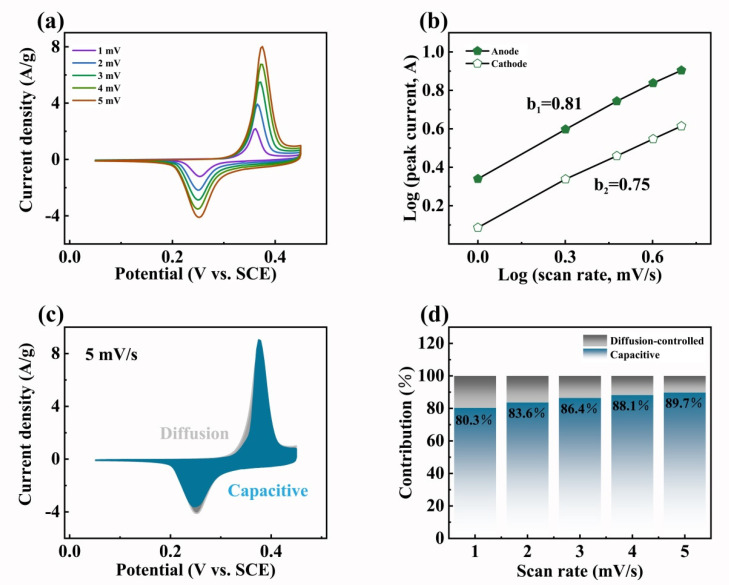
Analysis of the charge−storage mechanism of the NMH−2. (**a**) CV curves, (**b**) plot for diffusive and capacitive current contributions, (**c**) the contributions of capacitive− and diffusion−controlled processes at 5 mV/s, and (**d**) the fractions of capacitive and diffusion−controlled processes at different scan rates.

**Figure 8:**
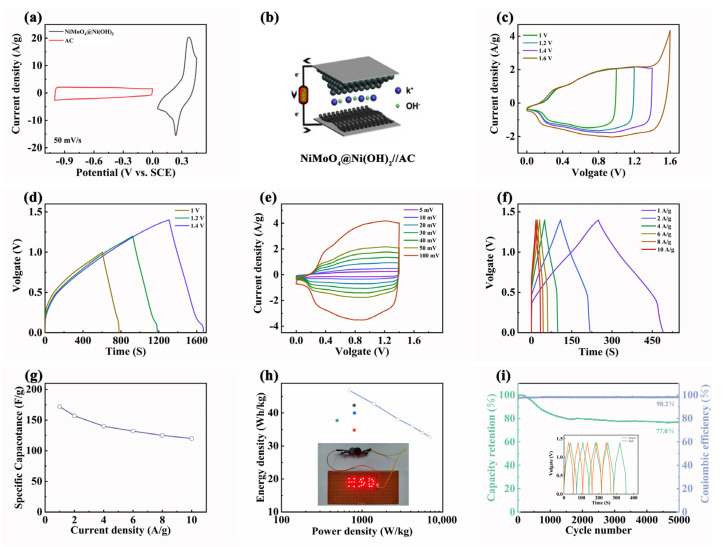
(**a**) CV curves of NMH−2 and AC in three−electrode systems. The energy storage performance of the NMH−2//AC. (**b**) the demonstration of the device. (**c**) CV curves under different working voltages at 50 mV/s. (**d**) GCD curves under various working voltages at 1 A/g. (**e**) CV curves at 5–100 mV/s. (**f**) GCD curves at 1–10 A/g. (**g**) Specific capacitances at 1–10 A/g. (**h**) Ragone plots. (**i**) Capacitance retention and Coulombic efficiency during cycling at 10 A/g.

In order to analyze the charge storage mechanism of NMH−2 and its possibility for practical applications, Figure 7a’s data were analyzed. A calculation based on the equation ($\;I={a\upsilon }^{b}$) [50] shows that there is a relationship between the peak current (i) and the scan rate (υ). The slopes (value of b) of the NMH−2 and AC electrodes (anode and cathode) are 0.81 and 0.75, respectively, indicating that the charge−storage mechanism of NMH−2 coexists with the processes of diffusion−control and capacitance [51,52]. Therefore, the above two contributions were studied separately. Figure 7c shows that the capacitive process provided most of the current response. As the scan rate increased to 5 mV s^−1^, the capacitive process proportion increased from 80.3% to 89.7% (Figure 7d). This phenomenon was caused by lagged access of electrolytes to active sites. To further expand the properties of the electrode material [53], NMH−2 and AC electrodes were used as asymmetric supercapacitor devices for the positive and negative electrode materials, and the appropriate potential window was selected in 2 M KOH for testing. During the assembly process, it was necessary to control the charge balance between the electrodes (q+ = q−), the mass ratio of NMH−2 to AC was fixed at 0.3 according to the formula, and the mass−loadings of NMH−2 and AC were 0.8 and 2.4 mg/cm^2^, respectively. Before testing the ASC device, we measured the CV curve of cathode NMH−2 and anode activated carbon at a sweep rate of 5 mV s^−1^ in a three−electrode system to estimate the best operating potential windows in Figure 8a. As shown in Figure 8b, the voltage window of the assembled ASC device can be up to approximately 1.4 V, approximately the sum of the voltages of two independent electrodes. Electrochemical test curves of Figure 8c,d show that the CV cycles and GCD trends of NMH−2//AC in various voltage windows confirm that the combined device can operate normally at 1.4 V with significant charge–discharge characteristics and little change in shape. After specifying the operating voltage of the most suitable device, as shown in Figure 8e, the CV diagram shows a shape close to a rectangle, but including a weak redox peak, which proves that the current response that makes up the device is contributed to by AC with a double−layer capacitance and NMH with a large pseudocapacitance value. The GCD curve of the assembled symmetrical supercapactior is almost linear, which further confirms the excellent fit of the selected cathode and anode materials (Figure 8f). The specific capacitance was evaluated against the GCD curve, and the current density increased from 1 A/g to 10 A/g. The capacitance values of NMH−2//AC (Figure 8g) were calculated as 172, 157,140, 125, and 121 F/g, respectively. Therefore, the energy density and power density of NMH−2//AC could be calculated, as shown in the Ragone plots (Figure 8h); furthermore, the NMH−2//AC could afford a high energy density of 46.8 Wh/kg at a power density of 700 W/kg, and this value is better than many NiMoO_4_−based ASCs, such as ZnCo_2_O_4_@Ni(OH)_2_//AC (40.0 Wh/kg at 802.7 W/kg) [47], Cu_2_O@Ni(OH)_2_//AC (34.8 W h/kg at 798 W/kg) [49], MnCo_2_O_4_@NiMoO_4_//AC (42.3 Wh/kg at 797 W/kg) [54], or AC//Ni(OH)_2_/expanded graphite (37.7 Wh/kg at 490.9 W/kg) [55]. To demonstrate the practical application of the NMH−2//AC device, the prototype device was connected to 23 small red LEDs and successfully lit them up (illustration in Figure 8h). As shown in Figure 8i, the NMH−2//AC exhibited an excellent cycling performance, maintaining 77.0% initial capacitance after 5000 cycles of 10 A/g and maintaining a Coulomb efficiency of 98.2% during this period.

## 4. Conclusions

The synthesis of NiMoO_4_@Ni(OH)_2_ nanofibers using simple strategies such as classical electrospinning, air calcination, and the one−step hydrothermal method, resulted in a large area of Ni(OH)_2_ nanosheets being grown on NiMoO_4_ nanofibers. Layered nanosheet Ni(OH)_2_ nanosheets can be grown on the surface of a one−dimensional (1D) material to expand the reaction area on which the optimal NMH−2 exhibits a high specific capacitance (1293 F/g at 1 A/g) and 56.8% of above−capacitance with a high current density 10 A/g) in the typical test condition. The assembled two−electrode asymmetric supercapacitor NMH−2//AC also shows a high energy density of 46.8 Wh/kg with a power density of 700 W/kg. The stability of the two−electrode asymmetric supercapacitor device is superior, maintaining 77.0% of virgin capacitance after the cycling test (5000 cycles). The Coulomb efficiency is calculated to reach 98.2%. This study proves the superiority of NiMoO_4_@Ni(OH)_2_ nanofibers as an energy−storage material and shows their unlimited potential in specific applications.

## Figures and Tables

**Table 1:** Elemental contents of the Mo, Ni, and O of NMO and NMH−1, 2, 3.

Element	Atomic %			
	NMO	NMH−1	NMH−2	NMH−3
O	72.75	77.40	72.30	64.28
Ni	12.03	12.12	15.24	18.92
Mo	15.22	10.48	12.46	16.80

**Table 2:** Comparison of specific capacitances of NMH−2 electrode materials with reported related electrodes at 1 A/g.

Supercapacitor	Specific Capacitance	Electrolyte	Ref.
NiMoO_4_ nanorods	815 F/g	3 M KOH	[5]
NiMoO_4_/rGO	706 F/g	3 M KOH	[45]
NiMoO_4_/NG	441 F/g	1 M KOH	[46]
ZnCo_2_O_4_@Ni(OH)_2_	1021 F/g	3 M KOH	[47]
CoMoO_4_@Ni(OH)_2_	1246 F/g	3 M KOH	[48]
Cu_2_O@Ni(OH)_2_	892 F/g	6 M KOH	[49]
This work	1293 F/g	2 M KOH	

## Data Availability

All of the relevant data are included in this published article.
